# Evaluation of community-based health promotion interventions in children and adolescents in high-income countries: a scoping review on strategies and methods used

**DOI:** 10.1186/s12889-023-15691-y

**Published:** 2023-05-10

**Authors:** Bettina Bader, Michaela Coenen, Julia Hummel, Petra Schoenweger, Stephan Voss, Caroline Jung-Sievers

**Affiliations:** 1 Institute for Medical Information Processing, Biometry, and Epidemiology – IBE, Chair of Public Health and Health Services Research, LMU Munich, Munich, Germany; 2 Pettenkofer School of Public Health, Munich, Germany

**Keywords:** Adolescent health, Child health, Community-based, Evaluation method, Evaluation strategy, Review, Study design

## Abstract

**Background:**

In recent decades, community-based interventions have been increasingly adopted in the field of health promotion and prevention. While their evaluation is relevant for health researchers, stakeholders and practitioners, conducting these evaluations is also challenging and there are no existing standards yet. The objective of this review is to scope peer-reviewed scientific publications on evaluation approaches used for community-based health promotion interventions. A special focus lies on children and adolescents’ prevention.

**Methods:**

A scoping review of the scientific literature was conducted by searching three bibliographic databases (Medline, EMBASE, PsycINFO). The search strategy encompassed search terms based on the PCC (Population, Concept, Context) scheme. Out of 6,402 identified hits, 44 articles were included in this review.

**Results:**

Out of the 44 articles eligible for this scoping review, the majority reported on studies conducted in the USA (*n* = 28), the UK (*n* = 6), Canada (*n* = 4) and Australia (*n* = 2). One study each was reported from Belgium, Denmark, Germany and Scotland, respectively. The included studies described interventions that mostly focused on obesity prevention, healthy nutrition promotion or well-being of children and adolescents. Nineteen articles included more than one evaluation design (e.g., process or outcome evaluation). Therefore, in total we identified 65 study designs within the scope of this review. Outcome evaluations often included randomized controlled trials (RCTs; 34.2%) or specific forms of RCTs (cluster RCTs; 9.8%) or quasi-experimental designs (26.8%). Process evaluation was mainly used in cohort (54.2%) and cross-sectional studies (33.3%). Only few articles used established evaluation frameworks or research concepts as a basis for the evaluation.

**Conclusion:**

Few studies presented comprehensive evaluation study protocols or approaches with different study designs in one paper. Therefore, holistic evaluation approaches were difficult to retrieve from the classical publication formats. However, these publications would be helpful to further guide public health evaluators, contribute to methodological discussions and to inform stakeholders in research and practice to make decisions based on evaluation results.

## Background

The field of health promotion and prevention has increasingly adopted community-based approaches over the past decades [1]. In addition to a wide range of meanings, the term ‘community-based’ can be defined as a setting, which is primarily geographical and is considered to be the place where interventions are carried out [2].

In the context of health promotion and prevention strategies, communities are highly relevant for planning and conducting interventions. Community-based approaches can enable access to target groups that are difficult to reach, such as people experiencing social disadvantages and people with existing health problems, without stigmatizing them in their daily lives [3].

Children and adolescents are an important target group in primary health promotion and prevention. If they come from families experiencing social disadvantages, they are not only more often exposed to health risks, but also less likely to benefit from health-related resources [4]. As communities are in position to change and adapt many health-related living conditions in different settings, they can play a key role in reaching this target group [4]. Therefore, health promotion measures can contribute to the reduction of socially determined inequalities in children's health opportunities and provide them with good development and participation perspectives regardless of their social status [4].

The latest approaches of community-based health promotion are determined by multiple components or complex interventions [5]. According to the Medical Research Council (MRC), an intervention is considered complex either because of the nature of the intervention itself or the “complex” way in which the intervention generates outcomes [6].

Evaluating complex interventions requires an appropriate set of methods to capture their different dimensions of effects, and to assess their impact and possible unintended consequences at the individual and societal levels. The key functions in evaluating complex interventions are assessing effectiveness, understanding change processes and implementation barriers and facilitators, and assessing cost-effectiveness [7]. Methodologically, we differentiate between process and outcome evaluations. Outcome evaluations on their own are often not sufficient or adequate to describe change in a system, but the process itself needs to be evaluated, such as the assessment of implementation fidelity and quality [7].

This understanding is important to implement interventions in a sustainable way, to describe processes such as empowerment and/or to justify policy and funding decisions. In addition, it allows future decisions and interventions to be further developed and improved. Achieving this goal requires comprehensive evaluation strategies and concepts with an elaborate set of combined methods, such as qualitative, quantitative and/or mixed methods within process and/or outcome evaluations [8, 9]. Theoretical evaluation frameworks, such as the RE-AIM framework [10] and others, can be used for planning and realizing evaluation approaches.

Although evaluation of public health interventions and more specifically community-based interventions is increasingly recognized as an important component of project conceptualization, implementation and management, published high quality methodology remains a major challenge [11].

To date, there are a range of strategies and concepts using a variety of qualitative and quantitative methods applied in different study designs to evaluate community-based interventions. To provide an overview and inform on current (good) practices, this scoping review aims at reporting on the strategies, concepts and methods used in studies evaluating community-based interventions focusing on health promotion and prevention in children and adolescents living in high-income countries.

## Methods

This scoping review was based on the framework by Arksey and O'Malley [12], which includes the following steps: identification of the research question and relevant studies, study selection, charting data, and collating, summarizing, and reporting results. The PRISMA Extension for scoping reviews (PRISMA-ScR) [13] and a pre-registered protocol were used as a guide in preparing the scoping review. The protocol was published in advance in the Open Science Framework and is accessible at the following link: https://osf.io/7vmah.

### Search strategy

To specify the search strategy, the categories of the PCC scheme (Population, Concept, Context) [14] were used and determinants were created based on the research question (Table 1).

**Table 1:** PCC framework

Category	Determinants
Population	Children and/or adolescents aged 0–19 years (according to WHO classification of adolescence^a^)
Concept	Evaluation strategies and concepts of health promotion and prevention interventions including study designs and methods
Context	Community-based

^a^ WHO, 2014 [15]

Three bibliographic databases were searched: Medline, EMBASE and PsycINFO. Keywords, truncations as well as limits to title and abstract fields were used for the database searches. The search strategy was adapted to the respective subcategories for each database, whereas the search terms remained identical. The following search strategy was used for the Medline and EMBASE databases: (child.tw. OR children.tw. OR teenager*.tw. OR youth.tw. OR adolescent*.tw.) AND (evaluat*.tw. OR monitor*tw.) AND (prevention.tw. OR health promotion/ OR health education/) AND (intervention.tw. OR program.tw. OR programme.tw. OR activit*.tw.) AND (communit*.tw. OR municipal*.tw. OR local.tw. OR neighbo?rhood.tw. OR rural.tw. OR urban.tw. OR district.tw.).

Database searches were conducted on April 8^th^, 2022. Only studies in English or German language were included. Due to the increase and further development of interventions in the community setting, the search was limited to publications from the last ten years (January 1^st^, 2012- until time of search).

The PCC framework [14] was used to establish the following inclusion and exclusion criteria (Table 2). Study design has been noted as an additional category.

**Table 2:** Inclusion and exclusion criteria

Category	Inclusion criteria	Exclusion criteria
Population	- Target population: Children and/or adolescents aged 0–19 years^a^	- Aged over 19 years - Specific subgroups (e.g., populations at risk)
Concept	- Evaluation strategies and concepts of health promotion and prevention interventions regarding a health outcome	- Evaluation strategies and concepts only focusing on cost-effectiveness or similar effects not involving health - COVID-19-related studies - Studies only focusing on digital environment and/or digitalization
Context	- Geographically-defined settings in the community-based, municipal or neighborhood sector - High income countries (classification according to the World Bank^b^)	- Actions and programs not considered to be community-based, or not in municipal or neighborhood settings - Institutional settings (e.g., health care or school settings) - Low and middle income countries (classification according to the World Bank^b^)
Study design	- Any empirical study design	- Non-empirical studies (e.g., commentaries or letters) - Reviews or meta-analyses

^a^ WHO, 2014 [15]; ^b^ World Bank, 2022 [16]

Studies in which the intervention also involved parents or other caregivers were taken into account, as long as the outcome primarily targeted children and adolescents.

We included only studies following a general population-based approach, i.e., offering interventions for the general population, not for specific risk populations [17]. Furthermore, we included only interventions in community settings and excluded school or hospital settings [18]. Institutional settings were only comprised if they were mentioned in addition to other settings and the intervention was not actively carried out there (e.g., recruitment through flyers posted at schools). Due to contact restrictions during the COVID-19 pandemic almost all community-based interventions were subject to profound adaptations (e.g., transition to digital offers). Since we wanted to provide a general overview of evaluation concepts not only restricted to pandemic circumstances, all COVID-19 related studies were excluded. Low- and middle-income countries were excluded because they face different circumstances, special target groups and different networking opportunities compared to high-income countries. Furthermore, reviews and meta-analyses were excluded, but their reference lists were checked to identify additional studies using the snowballing approach [19].

### Study selection

Search results were exported to the citation management software *Endnote*, where duplicates were removed. The study selection process was divided into two phases: (1) Title and abstract screening and (2) full text screening. The title and abstract screening was conducted by all authors using *Rayyan* [20], a web-based application that supports the initial title and screening process allowing the online collaboration of several researchers. To improve consistency between authors, all authors screened titles and abstracts of the same 100 publications, discussed the results and jointly adapted the guidance for the title and abstract screening, before starting the titles and abstract screening of all references.

For the full text screening, the included studies were integrated in an Excel spreadsheet available for all authors and validated by discussions with all authors. The same authors as in the title and abstract screening were involved in the full text screening process. In title and abstract as well as full text screening, 20% of the publications were reviewed independently by a second author. Discrepancies were discussed in the team and a decision was made collectively. In the title/abstract and the full text screening, publications were selected based on the predefined inclusion and exclusion criteria.

### Data extraction

Included studies were extracted using a customized spreadsheet. The data extraction was divided into two parts. The first part contained the general information such as first author, year of publication, country, population characteristics, setting, components, description of the intervention, frequency and duration of the intervention, presence of a comparison group, the objective(s) of the intervention and the type of article.

The second part comprised specific information focusing on the evaluation strategy, i.e., was the term process evaluation used (yes/no), was the term outcome evaluation used (yes/no), study type (observational/interventional), study design (randomized controlled trial (RCT)/cluster RCT/quasi-experimental/cross-sectional study/cohort study/case study) and the methods used (qualitative/quantitative/mixed methods). Furthermore, we extracted whether an evaluation framework, guidance or theoretical research approach was used.

We categorized studies as interventional if they were either experimental and/or quasi-experimental. If the study design was not explicitly mentioned, the categorization was done by the authors based on the methods section of the study.

The research team developed the data extraction sheet collaboratively. The first author (BB) independently extracted data from all included studies. Twenty percent were checked and extracted by a second author and discussed in the team to assess applicability and consistency of data extraction. Disagreements were discussed in the team and decisions were made collectively.

### Data synthesis

**Fig. 1:**
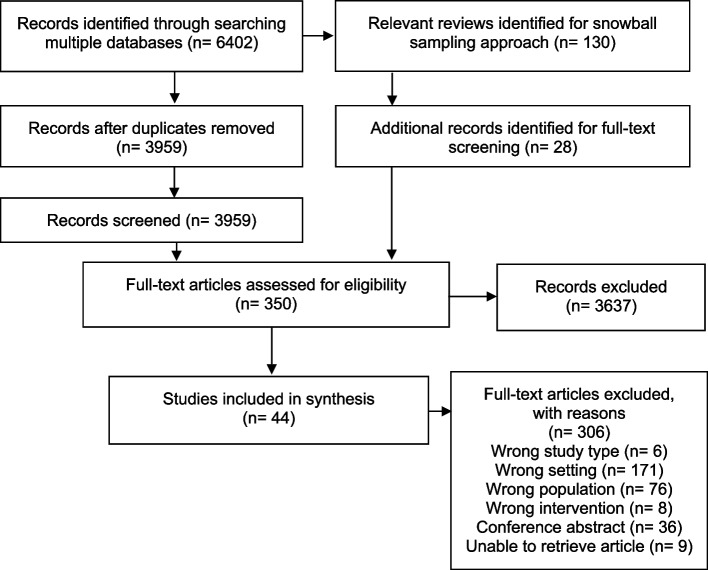
PRISMA-ScR flowchart of the screening process

The selection process was visualized by a PRISMA-ScR flowchart showing the results of the screening steps (Fig. 1). The results of the data extraction were presented in tables and as a narrative summary.

## Results

A total of 6,402 articles were identified from searching the three bibliographic databases after the removal of duplicates, 3,959 publications remained for the title and abstract screening. A total of 130 reviews were identified in the literature search of which 20 were considered as relevant for our research question. From these reviews, 28 additional studies were eligible for the full text screening [21–40].

A total of 350 articles were included for full text screening and assessed for eligibility. Here, 44 studies met our inclusion criteria, while 306 were excluded. The most prevalent exclusion criterion was the wrong setting (*n* = 171). This criterion was applied, for instance, if the whole or a part of the intervention took place in an institutional setting such as schools. Another exclusion criterion was “wrong population” (*n* = 76). Examples for this criterion to be applied was: children were not involved in the intervention, although the outcome might have targeted them. Other exclusion criteria were wrong study type (e.g., non-empirical studies, *n* = 6), wrong intervention (*n* = 8); lack of health-related outcome; no full text available (conference abstracts (*n* = 36)), and no accessibility to the full article (*n* = 9). If there was more than one reason for exclusion, the final reason was chosen according to the following hierarchy: 1) wrong study type, 2) wrong setting, 3) wrong population and 4) wrong intervention.

## Characteristics of included studies

Table 3 provides an overview of included studies. Included articles were published between 2012 and 2022, and addressed either primary studies with research findings (*n* = 38) or published research protocols (*n* = 6). The studies were mainly conducted in the USA (*n* = 28; 63.6%), followed by the UK (*n* = 6; 13.6%), Canada (*n* = 4; 9.1%), Australia (*n* = 2; 4.6%), Belgium (*n* = 1; 2.2%), Denmark (*n* = 1; 2.2%), Germany (*n* = 1; 2.2%) and Scotland (*n* = 1; 2.2%).

**Table 3:** Identified studies

AUTHORS	YEAR PUBLISHED	COUNTRY	STUDY TYPE	EVALUATION STRATEGIE(S)	STUDY DESIGN(S)	POPULATION AGE (YEARS)	INTERVENTION
ABEBE ET AL. [41]	2018	USA	Interventional	a) Outcome evaluation b) Process evaluation	a) Cluster RCT b) Cohort study	13–19	Sexual violence and/or adolescent relationship abuse prevention
BELL ET AL. [42]	2019	Australia	Interventional	a) Outcome evaluation	a) Quasi-experimental	MA^a^ 10.6	Obesity prevention; Healthy nutrition promotion; Well-being
BERGE ET AL. [43]	2016	USA	Interventional	a) Outcome evaluation b) Process evaluation	a) Quasi-experimental b) Cross-sectional	6–12	Obesity prevention
BOTTORFF ET AL. [44]	2020	Canada	Observational	a) Outcome evaluation b) Process evaluation	a) Cross-sectional b) Cross-sectional	0–18	Obesity prevention; Healthy nutrition prevention; Well-being
BOTTORFF ET AL. [45]	2021	Canada	Observational	a) Outcome evaluation b) Process evaluation	a) Cohort study b) Cohort study	0–18	Obesity prevention; Healthy nutrition prevention; Well-being
BROPHY-HERB ET AL. [46]	2017	USA	Interventional	a) Outcome evaluation b) Process evaluation	a) RCT b) Cohort study	3–5	Obesity prevention
BROWN ET AL. [47]	2021	USA	Interventional	a) Outcome evaluation	a) Cluster RCT	13–19	Pregnancy prevention
DANNEFER ET AL. [48]	2016	USA	Observational	a) Outcome evaluation	a) Cross-sectional	2–15	Healthy nutrition promotion
EXNER-CORTENS ET AL. [49]	2020	Canada	Interventional	a) Outcome evaluation b) Process evaluation	a) RCT b) Cohort study	MA 15.5	Problem behavior prevention^b^; Sexual violence and/or adolescent relationship abuse prevention
FAIR ET AL. [50]	2017	USA	Interventional	a) Outcome evaluation	a) Quasi-experimental	0–18	Obesity prevention
FLATTUM ET AL. [51]	2015	USA	Interventional	a) Outcome evaluation b) Process evaluation	a) RCT b) Cohort study	8–12	Obesity prevention; Healthy nutrition promotion
GARCIA ET AL. [52]	2020	Scotland	Observational	a) Outcome evaluation	a) Cohort study	0–4	Healthy nutrition promotion
GILLESPIE ET AL. [53]	2019	Scotland	Interventional	a) Outcome evaluation b) Process evaluation	a) RCT b) Cross-sectional	2.5–5	Obesity prevention; Healthy nutrition promotion
GITTELSOHN ET AL. [54]	2013	USA	Observational	a) Outcome evaluation b) Process evaluation	a) RCT b) Cross-sectional (repeated)	10–14	Obesity prevention; Healthy nutrition promotion
GITTELSOHN ET AL. [55]	2017	USA	Interventional	a) Outcome evaluation	a) RCT	10–14	Obesity prevention; Healthy nutrition promotion
GRIER ET AL. [56]	2015	USA	Observational	b) Process evaluation	b) Cohort study	MA 8.7	Obesity prevention; Healthy nutrition promotion
HILL ET AL. [57]	2022	USA	Interventional	a) Outcome evaluation b) Process evaluation	a) Quasi-experimental b) Cohort study	13–19	Sexual violence and/or adolescent relationship abuse prevention; Sexual health promotion
HOFFMAN ET AL. [58]	2014	USA	Observational	a) Outcome evaluation	a) Cross-sectional (repeated)	≤ 18	Oral health promotion
HOLLAND ET AL. [59]	2015	USA	Observational	a) Outcome evaluation b) Process evaluation	a) Cohort study b) Cohort study	n.o.^c^	Problem behavior prevention
IACHINI ET AL. [60]	2014	USA	Observational	b) Process evaluation	b) Cohort study	MA 16.1	Problem behavior prevention; Obesity prevention
JACOBS ET AL. [61]	2021	Australia	Interventional	a) Outcome evaluation	a) Quasi-experimental	6–13	Obesity prevention; Healthy nutrition promotion
JUNG ET AL. [62]	2018	Canada	Observational	a) Outcome evaluation b) Process evaluation	a) Cohort study b) Cohort study	MA 13	Obesity prevention; Healthy nutrition prevention; Well-being
MAITLAND ET AL. [63]	2019	Australia	Observational	a) Outcome evaluation	a) Cohort study	5–12	Obesity prevention
MATHEWS ET AL. [64]	2018	USA	Interventional	a) Outcome evaluation	a) RCT; quasi-experimental	9–10	Obesity prevention; Healthy nutrition promotion
MCINTOSH ET AL. [65]	2015	Canada	Observational	b) Process evaluation	b) Case study	n.o.	Obesity prevention
MILLER ET AL. [66]	2020	USA	Interventional	a) Outcome evaluation	a) Cluster RCT	13–19	Sexual violence and/or adolescent relationship abuse prevention
MORRISON BEEDY ET AL. [67]	2013	USA	Interventional	a) Outcome evaluation	a) RCT	15–19	Sexual health promotion
OTTO ET AL. [68]	2020	USA	Interventional	a) Outcome evaluation	a) RCT	12–16	Smoking prevention
OVERCASH ET AL. [69]	2018	USA	Observational	a) Outcome evaluation	a) Cohort study	9–12	Healthy nutrition promotion
PAWLOWSKI ET AL. [70]	2017	Denmark	Interventional	a) Outcome evaluation b) Process evaluation	a) Quasi-experimental b) Cohort study	10–13	Obesity prevention; Active living promotion
RHEW ET AL. [71]	2016	USA	Interventional	a) Outcome evaluation	a) Quasi-experimental	MA 11.6- 15.6	Problem behavior prevention
ROBERTSON ET AL. [72]	2016	UK	Observational	a) Outcome evaluation b) Process evaluation	a) Cross-sectional b) Cross-sectional	1–16	Well-being
ROBINSON ET AL. [73]	2016	USA	Interventional	a) Outcome evaluation b) Process evaluation	a) RCT; cluster RCT b) Cohort study	MA 13.9 MA 12.3	Problem behavior prevention; Pregnancy prevention
RÖDING ET AL. [74]	2021	Germany	Interventional	a) Outcome evaluation b) Process evaluation	a) Quasi-experimental b) Cross-sectional	11–17	Problem behavior prevention; Well-being
SALAZAR ET AL. [75]	2016	USA	Interventional	b) Process evaluation	b) Quasi-experimental	n.o.	Prevention of child maltreatment; Well-being
SALAZAR ET AL. [76]	2019	USA	Interventional	b) Process evaluation	b) Quasi-experimental	n.o.	Prevention of child maltreatment; Well-being
SEIRAWAN ET AL. [77]	2021	USA	Interventional	a) Outcome evaluation	a) RCT	0–5	Oral health promotion
SKOUTERIS ET AL. [78]	2016	Australia	Interventional	a) Outcome evaluation	a) RCT	MA 2.7	Obesity prevention; Healthy nutrition promotion
SMITH ET AL. [79]	2019	Canada	Observational	a) Outcome evaluation	a) Cohort study	MA 9.8	Obesity prevention
STRUNIN ET AL. [80]	2013	USA	Observational	b) Process evaluation	b) Cross-sectional	13–18	Problem behavior prevention; Healthy nutrition promotion
TRUDE ET AL. [81]	2018	USA	Interventional	a) Outcome evaluation b) Process evaluation	a) RCT b) Cross-sectional	n.o.	Healthy nutrition promotion
UMSTATTD MEYER ET AL. [82]	2019	USA	Observational	a) Outcome evaluation	a) Cross-sectional	3–15	Obesity prevention
VINCK ET AL. [83]	2016	Belgium	Interventional	a) Outcome evaluation	a) Quasi-experimental	MA 4.8–4.9	Obesity prevention
WHITE ET AL. [84]	2019	USA	Interventional	a) Outcome evaluation b) Process evaluation	a) RCT; quasi-experimental b) Cohort study	9–10	Obesity prevention; Healthy nutrition promotion

^a^MA: Mean age; if there was no information about the age range of the population but on the mean age, this information has been added to this column

^b^Problem behavior prevention includes prevention of antisocial behavior, substance use, violence, and delinquency

^c^n.o. implies that the information was not obtainable

Among the children and adolescents examined in the included studies, age ranged from 0 to 19 years, and the population often received the intervention as families or parent-child dyads.

In most cases, interventions mainly aimed at: obesity prevention (*n* = 23; 52.3%), healthy nutrition promotion (*n* = 15; 34.1%), well-being (*n* = 9; 20.5%), problematic behavior prevention (including antisocial behavior, substance use, violence, delinquency; *n* = 7; 15.9%) or sexual violence and/or adolescent relationship abuse prevention (*n* = 4; 9.1%). Five interventions were reported in more than one of the included studies. Thus, 36 different interventions were represented in the 44 included studies. The studies used different study designs such as observational study designs (*n* = 17) and interventional study designs (*n* = 27).

## Strategies and methods of evaluation

Of the 44 articles included, nearly half of them aimed at evaluating outcomes only (*n* = 20; 45.5%) [42, 47, 48, 50, 52, 55, 58, 61, 63, 64, 66–69, 71, 77–79, 82, 83], whereas 18 described outcome and process evaluation (40.9%) [41, 43–46, 49, 51, 53, 54, 57, 59, 62, 70, 72–74, 81, 84], and 6 focused on process evaluation solely (*n* = 6; 13.6%) (56, 60, 65,75, 76, 80). However, only a few studies explicitly used the terms ‘process evaluation’ (*n* = 14) and/or ‘outcome evaluation’ (*n* = 4) to describe their evaluation strategies.

A total of 19 studies presented more than one method used for evaluation (e.g., cross-sectional study and RCT applied within one study). Therefore, this review identified 65 study designs within different evaluation classifications (Table 4).

**Table 4:** Study designs stratified by evaluation strategies

**Evaluation concept**		**Outcome evaluation**	**Outcome evaluation incl. process evaluation**	**Process evaluation**
	**N**	**N (%**^**a**^**)**	**N (%**^**b**^**)**	**N (%**^**c**^**)**
**RCT**	14	14 (34.2%)	8 (57.1%)	0 (0.0%)
**Cluster RCT**	4	4 (9.8%)	2 (50.0%)	0 (0.0%)
**Quasi-experimental**	13	11 (26.8%)	5 (45.5%)	2 (8.3%)
**Cohort study**	20	7 (17.1%)	3 (42.9%)	13 (54.2%)
**Cross-sectional**	13	5 (12.2%)	2 (40.0%)	8 (33.3%)
**Case study**	1	0 (0.0%)	0 (0.0%)	1 (4.2%)

^a^ n/ number of study designs using strategies of outcome evaluation (*N* = 41)

^b^ n/ number of such type of designs (e.g.: 57.1% of RCTs included process evaluation)

^c^ n/ number of study designs using strategies of process evaluation (*N* = 24)

Studies reporting on outcome evaluations often applied RCTs (34.2%), specific forms of RCTs (such as cluster RCTs; 9.8%) or quasi-experimental designs (26.8%). Other study designs for outcome evaluation strategies were observational study designs, such as cohort studies (17.1%) or cross-sectional studies (12.2%; almost half of them used a repeated cross-sectional design). Process evaluation strategies were described in 48.8% (*n* = 20) of the included studies (53.6% (*n* = 15) within interventional designs and 38.5% (*n* = 5) within observational designs). Process evaluation used mainly cohort (54.2%) and cross-sectional study designs (33.3%); one out of 8 used a repeated cross-sectional study design. Two quasi-experimental and one case study were included.

In terms of methods used, in 25 publications including 33 different study designs quantitative methods were reported (*n* [41, 46–50, 54, 55, 58, 61, 64, 66–69, 71, 75–79, 81–84], 16 publications reported on 27 mixed method designs (*n*) [42–45, 51–53, 56, 57, 60, 62, 63, 70, 73, 74, 80] and 3 publications reported on 5 qualitative methods based designs [59, 65, 72] (Table 5).

**Table 5:** Individual study designs (*N* = 65) reported within the 44 publications stratified by methods used

Study design	Total	Quantitative	Qualitative	Mixed methods
	**N**	**N (%**^**a**^**)**	**N (%**^**a**^**)**	**N (%**^**a**^**)**
**RCT**	14	11 (16.9%)	0 (0.0%)	3 (4.6%)
**Cluster RCT**	4	3 (4.6%)	0 (0.0%)	1 (1.6%)
**Quasi-experimental**	13	8 (12.3%)	0 (0.0%)	5 (7.7%)
**Cohort study**	20	6 (9.2%)	2 (3.1%)	12 (18.5%)
**Cross-sectional**	13	5 (7.7%)	2 (3.1%)	6 (9.2%)
**Case study**	1	0 (0.0%)	1 (1.5%)	0 (0.0%)
**Total**	65	33 (50.8%)	5 (7.7%)	27 (41.5%)

^a^ n/ number of designs (*N* = 65)

### Reference to frameworks, theories or guidance of included studies

Few studies referred to frameworks or guidelines that provide a basis for evaluation. Bottorff et al. [44, 45] and Jung et al. [62] referred to the RE-AIM framework (RE-AIM = reach, effectiveness, adoption, implementation and maintenance), which is an evidence-based framework developed for assessing the real-world applicability and effectiveness of health-related interventions in community settings [10, 44].

The RE-AIM framework was used for outcome and process evaluation and focused on the five established dimensions: reach, effectiveness, adoption, implementation and maintenance. Both studies referring to RE-AIM were examining the same intervention from different perspectives. They each conducted an observational study – i.e. cross-sectional and cohort study.

Other authors such as Gillespie et al. referred to the MRC guidance [53]. They presented both outcome and process evaluation in their study protocol for a RCT. They planned to use a logic model with three phases: participatory methods (phase 1), recruitment, consent, randomization (phase 2), and intervention trial (phase 3). Each phase included the following dimensions: activities, reach, short-term outcomes, intermediate outcomes and long-term outcomes. They designed their process analysis according to the MRC guideline for process evaluation [85]. Within phase 1 and 2, data will be evaluated in terms of participatory, co-productive approach and possible adjustments to the original design or methods. In phase 3, components of implementation will be considered such as context, feasibility and acceptability.

Several of the identified studies focused on the same intervention approach: four studies focused on the Communities that Care (CTC) approach – a scientific approach to address problem behaviors in children and adolescents on a community level. The CTC approach consists of 5 phases: assess community readiness, get organized at community level, develop a community profile, select and implement suitable evidence-based programs. For each phase, there is a detailed task description and a tool for self-reporting the benchmarks achieved.

To evaluate CTC approaches, Rhew et al. [71], Röding et al. [74] and Salazar et al. [75, 76] used quasi-experimental designs with other communities as comparison groups. The last two groups of authors also integrated a logic model.

The Community-Based Participatory Research (CBPR) approach was applied in four of the included studies. CBPR is defined as a collaborative effort and equal partnering of all stages of the research process between researchers and community members and organizations to meet the needs of the community [86]. Berge et al. [43] used a quasi-experimental design for the implementation of the intervention and a cross-sectional design for the process evaluation. The core principles of the CBPR approach were described and the authors used the theoretical Citizen Health Care Model, a CBPR approach, to guide the study design as well as hypothesis development and analyses. Grier et al. [56] and McIntosh et al. [65] used observational designs for their process evaluation and showed a positive response from community members through the collaborative approach. White et al. [84] conducted interventional studies and demonstrated both process and outcome evaluation, using the CBPR approach as the structure of their study.

## Discussion

In this review, we scoped the existing literature on evaluation strategies, study designs, concepts and methods used for community-based health promotion and prevention interventions targeting children and adolescents. Overall, we included 44 studies based on our predefined search criteria and identified a total of 65 evaluation designs used in these studies. We identified different evaluation strategies and methods that have been used in this research field. Our main results were i) a content related focus of studies reporting on the evaluation of intervention targeting obesity and nutrition, ii) a methodological imbalance and focus on outcome evaluation strategies with RCTs and/or quasi-experimental designs to the disadvantage of process evaluation strategies and qualitative methods in the included studies, and iii) a lack of application of or referral to consistent standards, guidance or methods for the design of evaluation strategies.

i) Aim of interventions

The majority of the studies focused, among others, on the prevention of obesity and were also often linked to the promotion of healthy nutrition. This may be due to the fact that obesity, defined as “abnormal or excessive fat accumulation that may impair health” [87] by the World Health Organization (WHO) is considered one of the most prevalent health issues facing children and adolescents worldwide [87]. Although there is evidence that obesity rates are stagnating or decreasing in many high-income countries [88], it is still a relevant issue as the numbers remain high [89].

Community-based interventions, combined with population-wide interventions (e.g., social marketing campaigns), and structural modifications (e.g., establishment of networks and partnerships), are recommended by the WHO as an effective and long-lasting way to prevent childhood obesity [90]. As our review focuses on the community setting, this may also reflect a reason for the large proportion of these health prevention interventions in the included studies.

Other areas that have emerged in our research were the prevention of problem behavior and the prevention of sexual violence and/or relationship abuse among adolescents. Problematic behavior included issues such as antisocial behavior, substance use, violence or delinquency. The relevance of these fields could be explained by the fact that, in general, adolescence is a period with an increased susceptibility to risky behavior [49]. At the same time, this phase in life can also be characterized by the development of positive values and skills [49].

ii) Evaluation strategies: Outcome and process evaluation

A range of study designs are available and each design is differently suited to answer different research questions. Our research reveals two areas in which different study designs were preferred for evaluation: outcome evaluation and process evaluation. Based on our results, it seems like the focus is still mainly on outcome evaluation, as most of the publications referred to outcome evaluations and only half of them integrated process evaluation strategies. A preponderance of outcome evaluations may be due to the current general dominance of this methodology in science, as it is often solely about effectiveness and effects are often measured in numbers. However, process evaluation strategies are equally relevant as they help to understand why a program has been successful or not. Applying process evaluation strategies is also of utmost importance to guide and support the process of implementing and adapt interventions and finally to facilitate the consolidation and sustainability of interventions in the community.

Furthermore, the terms 'process evaluation' and 'outcome evaluation' were hardly used in studies included in our review (the former, however, more often). Nevertheless, only few studies could be included that dealt exclusively with process evaluation. This may be due to our own methodology and is referred to in the ‘Strengths and Limitations’ section. Studies dealing with outcome evaluations were also integrating process evaluation or the wording quite rare.

It is questionable whether this is caused by the fact that studies dealing with outcome evaluations have not conducted any process evaluation strategies at all. One alternative explanation could be a publication bias favoring quantitative studies over qualitative or the fact that health care researchers are not yet familiar with the concepts of process and outcome evaluation. On the other hand, using the term ‘outcome evaluation’ does not seem to be common practice in the research field of studying the effectiveness of an intervention, or the researchers involved may not always be aware that they are conducting an outcome evaluation. This reflects the need to disseminate these evaluation strategies and methods more widely among scientists to obtain comprehensive evaluations using both strategies and applying different quantitative and qualitative methods in the future.

In the context of outcome evaluations, the focus was primarily on interventional studies such as RCTs (including adaptions such as cluster RCTs) and quasi-experimental studies, respectively. RCTs are known for their ability to verify the cause-effect relationship between an intervention and an outcome, and are therefore the gold standard for evaluating effectiveness [91]. Despite their high level of evidence, they may also often not be feasible or adequate in the area or setting of community-based health promotion interventions. RCTs can be particularly limited when the context of implementation essentially affects the outcome. The conditions in an experiment may differ from those in real life and the results may not apply in a non-experimental setting [39, 92, 93]. In order to improve the impact of complex intervention research, standard designs such as RCTs need to be further developed and adapted to suit complex intervention contexts according to the MRC guidance [7, 93]. In our review only four studies used an adaptation of this study design, i.e. cluster RCTs.

Especially in community settings, such study designs are feasible and valuable for conducting interventions at group levels and/or avoiding potential contamination between groups [94]. Robinson et al. [73] conducted their study in two community-based settings. They used one RCT and one cluster RCT and reported that in both trials, no impact on the outcome was demonstrated by the intervention. The reason for the different study designs was neither addressed nor discussed in the study. However, this would have been an important and interesting point of discussion. In practice, it may not be possible to randomly distribute the intervention due to practical or ethical reasons. Especially in the context of a community, it may not be feasible for only half of the people or sites to receive an intervention. This could lead to spillover effects, underestimating the overall benefit of the intervention for the target population [95].

Due to a growing interest in comparative effectiveness studies and the raising relevance of external validity, quasi-experimental and non-experimental studies have received increased attention in the field of public health. Natural and quasi-experimental approaches provide the opportunity to access changes in a system that would be difficult to influence through experimental designs [96]. Especially in community-based interventions, environmental changes are often added as part of the intervention as seen in this review. Due to the combination of characteristics of experimental and non-experimental designs, quasi-experimental studies can cover such interventions and their evaluation. Quasi-experiments usually use data on other entire population groups [97]. In the study of Bell et al. included in this review, 20 matched communities were used as control groups for the outcome evaluation [42]. Data is usually collected using routine data systems such as clinical records or census data [97]. However, a common criticism of quasi-experimental studies is that the processes leading to variations are beyond the control of the studies [98]. Therefore, it impossible to determine whether confounding has been successfully prevented [98].

Quantitative methods were used in 11 of 14 RCTs and 3 of 4 cluster RCTs within the studies reported here. As these study designs can specifically demonstrate effectiveness and causal associations, the choice of methodology is appropriate for outcome evaluations. However, a purely quantitative approach within a RCT without additional components such as process analysis is hardly suitable for the evaluation of complex interventions according to MRC guidance [93]. Quite often, it will be necessary to answer those questions that go beyond effectiveness. Qualitative or mixed methods are more appropriate for this purpose [93] as qualitative research may give insights into subjective views and perceptions of individuals and stakeholders. In process evaluation studies in this review, we were able to identify mainly observational designs such as cross-sectional or cohort studies. Although guidelines exist and process evaluations are carried out in principle [99], the methodological variety available for process evaluation strategies do not seem to be exploited to its full potential to date. We hypothesize that there is room for development here, since qualitative methods are suitable to elaborate important indications why an intervention is (or is not) working and how it could be improved. In addition, the iterative nature of data collection and interpretation in qualitative methods support participatory adaptation of the intervention and knowledge translation into the field of interest during implementation. Or vice versa, if qualitative methods are not incorporated into evaluation strategies, important findings may be missed and possible new hypotheses, views and developments may not be recognized and noted.

iii) Concepts and guidelines

Only a few studies were found in this review that explicitly referred to guidelines, frameworks or similar concepts for evaluation [44, 45, 62]. Among those, RE-AIM offers an efficient framework and thus provides a systematic structure for both planning and evaluating health-related interventions [85]. Jung et al. [62] provided a precise overview of their steps and each of the five dimensions of the framework were evaluated individually using a mixed-methods study design. It was also described that mainly quantitative methods were used to examine outcomes. For the process evaluation, on the other hand, more qualitative methods should be used to get rich and meaningful data. This kind of data could be used to guide the conceptualization and implementation of complex interventions. Bottorff et al. [45] described the RE-AIM framework as another feasible option to collect information to guide planning for future scale-up. In this sense, it offers a robust concept to guide evaluation approaches. Limitations of the evaluation remain, however, due to the difficulty of balancing a scientifically relevant evaluation with the needs of the study participants through appropriate assessment instruments [45].

Other pragmatic guidance is offered by the MRC framework, the CTC approach and principles of CBPR. Gillespie et al. [53], for instance, included both a logic model and the MRC's guidance for process evaluations in their evaluation concept. The MRC recommends a framework based on the themes of implementation, mechanisms and context. The guidance provides an overview of key recommendations regarding planning, design and implementation, analysis and reporting, and suggests, among other things, the use of a logic model to clarify causal assumptions [99].

Another approach used in this review was the CTC approach [71, 74–76]. This framework was originally developed in the US to guide community coalitions in planning and implementing community-based health promotion interventions targeting children and adolescents [100]. This is primarily an implementation plan, but also provides information on quality assessment and further development. CTC offers evaluation tools and supports the implementation process [74]. CTC is more common in the USA but is increasingly being used in other countries. Although evidence and tools for the process of CTC are provided, precise concepts for outcome evaluation seem to be lacking here as well.

Principles of CBPR were also used in four studies [43, 56, 65, 84]. These focused mainly on process evaluation, which reflects the relevance of this evaluation approach, as it is particularly important to ascertain whether the intervention is accepted by all participants in a collaborative environment. The CBPR approach provides structure for developing and implementing interventions, but also includes approaches for evaluating processes and outcomes. CBPR projects are characterized by several core principles that are designed to enable and strengthen the collaborative approach, and focus on action and participation of all stakeholders [101]. Due to individual research questions and contexts of each partnership, it is impossible to prescribe a design to be used; rather, each must determine for itself what is most appropriate [101]. The principles serve as a guideline and support to develop own structures. Through the collaborative process, data can be collected that accurately reflects the real-world experiences of the members [102]. Berge et al. [43] demonstrated that using the CBPR framework, researchers and community members collaboratively developed an intervention, and results showed high participant satisfaction in addition to high feasibility. Although there are also concrete logical models of the CBPR approach that give clear structures about contexts, group dynamics, interventions and outcomes [103], these were not integrated in the studies of this review.

### Strengths and limitations

As with any project, the chosen approach, design and methodology has several limitations as well as strengths. One limitation was that we potentially missed out some studies or study designs by applying the defined search strategy which 1) was limited to the last 10 years, 2) only included sources published in the English or German language, 3) used specific search terms narrowed by the PCC scheme, and 4) was only conducted in three databases. The latter aspect could have led to the fact that studies using process evaluation strategies may have been underrepresented, as the selected databases may be very medically and quantitatively loaded. Another limitation of the work was the exclusion of interventions conducted in institutional setting such as schools. This setting plays an important role in health promotion for children and adolescents. It particularly offers practical opportunities for the implementation of comprehensive strategies, but was not explored due to the institutional approach with different characteristics than a purely community-based approach. Therefore, our understanding of community based approaches was narrow by nature. A methodological challenge, especially during the screening process, was the heterogeneity and equivocality of the terminology used for community-based health promotion and prevention projects. For future projects, a broader approach and scope of the review, including additional keywords and databases for the search strategy, could be considered. Additionally, databases that focus on other subject areas such as education sciences or social sciences could provide more results with regard to studies using process evaluation strategies.

A key strength of the scoping review was the sound methodology based on the framework recommended by Arksey and O'Malley and the PRISMA-ScR checklist. Furthermore, the collaborative approach, as well as the 20% double screening in each of the screening and data extraction phases as well as regular team discussions in all stages of the project, ensured consistency, feasibility and thus a high level of quality. The review provides an overview of selected study designs and methodologies for future research. While there is no clear recommended approach, researchers can use our review to guide future interventions and get suggestions for evaluation concepts and strategies.

## Conclusion

In our scoping review, we identified important trends in the field of health prevention and promotion evaluations of children and adolescents in high-income countries. Although a variety of different methods and approaches exist, the choice of methods to evaluate community-based interventions depends on various factors. Guidance to inform approaches can be drawn from RE-AIM, the CTC and CBPR concepts. The widely used and recently updated MRC framework indicates that evaluation is moving beyond questions of effectiveness and is therefore leading to a change in research priority, which includes the importance of process evaluation and qualitative methods [93]. Increasing attention will be paid to whether and how the acceptability, feasibility and transferability of an intervention can be obtained in different settings or contexts [93]. As evaluation concepts and strategies are complex with a wide range of contexts and methods to consider, it would be helpful to expand publication strategies on the evaluation of complex interventions to further guide public health experts and scientists, to contribute to methodological discussions and to make informed and evidence-based decisions based on evaluation results.

## Data Availability

The protocol is published in OSF. Additional information is available upon request to the corresponding author (CJS).

## Abbreviations

CBPR: Community-based participatory research approach
CTC: Communities That Care
MRC: Medical Research Council
PCC: Population, Concept, Context
PRISMA‐ScR: PRISMA Extension for Scoping Reviews
RCT: Randomized controlled trials
WHO: World Health Organization
